# Laboratory Diagnostics Market in East Africa: A Survey of Test Types, Test Availability, and Test Prices in Kampala, Uganda

**DOI:** 10.1371/journal.pone.0134578

**Published:** 2015-07-30

**Authors:** Lee F. Schroeder, Ali Elbireer, J. Brooks Jackson, Timothy K. Amukele

**Affiliations:** 1 Department of Pathology, University of Michigan School of Medicine, Ann Arbor, Michigan, United States of America; 2 Makerere University-Johns Hopkins University Clinical Core Laboratory at Infectious Diseases Institute, Kampala, Uganda; 3 Department of Medicine, Johns Hopkins University School of Medicine, Baltimore, Maryland, United States of America; 4 Department of Laboratory Medicine and Pathology, University of Minnesota Medical School, Minneapolis, Minnesota, United States of America; 5 Department of Pathology, Johns Hopkins University School of Medicine, Baltimore, Maryland, United States of America; McGill University, CANADA

## Abstract

**Background:**

Diagnostic laboratory tests are routinely defined in terms of their sensitivity, specificity, and ease of use. But the actual clinical impact of a diagnostic test also depends on its availability and price. This is especially true in resource-limited settings such as sub-Saharan Africa. We present a first-of-its-kind report of diagnostic test types, availability, and prices in Kampala, Uganda.

**Methods:**

Test types (identity) and availability were based on menus and volumes obtained from clinical laboratories in late 2011 in Kampala using a standard questionnaire. As a measure of test availability, we used the *Availability Index (AI)*. AI is the combined daily testing volumes of laboratories offering a given test, divided by the combined daily testing volumes of all laboratories in Kampala. Test prices were based on a sampling of prices collected in person and via telephone surveys in 2015.

**Findings:**

Test volumes and menus were obtained for 95% (907/954) of laboratories in Kampala city. These 907 laboratories offered 100 different test types. The ten most commonly offered tests in decreasing order were Malaria, HCG, HIV serology, Syphilis, Typhoid, Urinalysis, Brucellosis, Stool Analysis, Glucose, and ABO/Rh. In terms of AI, the 100 tests clustered into three groups: high (12 tests), moderate (33 tests), and minimal (55 tests) availability. 50% and 36% of overall availability was provided through private and public laboratories, respectively. Point-of-care laboratories contributed 35% to the AI of high availability tests, but only 6% to the AI of the other tests. The mean price of the most commonly offered test types was $2.62 (range $1.83–$3.46).

**Interpretation:**

One hundred different laboratory test types were in use in Kampala in late 2011. Both public and private laboratories were critical to test availability. The tests offered in point-of-care laboratories tended to be the most available tests. Prices of the most common tests ranged from $1.83-$3.46.

## Introduction

The impact of a laboratory test on health outcomes depends on characteristics that are intrinsic to the test such as its diagnostic accuracy, complexity, and result interpretability. It also depends on characteristics that are extrinsic to the test such as commercial demand, price, and availability.[1,2] These extrinsic characteristics are described through needs assessments and utilization reviews. Such assessments and reviews have been ongoing for human immunodeficiency virus infection / acquired immune deficiency syndrome (HIV/AIDS), tuberculosis, and malaria.[3–5] For example, landscape reports for HIV, TB, and malaria are regularly updated by UNITAID.[6–8] But this narrow focus does not reflect the total disease burden in sub-Saharan Africa.

According to estimates of the Institute for Health Metrics and Evaluation (IHME), HIV, TB, and malaria accounted for 29% of deaths in this region in 2010.[9,10] Similarly, the United Nations General Assembly High Level Meeting on Non-communicable Diseases highlighted the rapidly growing prevalence of cardiovascular disease, cancer, respiratory disorders, and diabetes in developing nations.[11] Thus in SSA, there is a need for comprehensive diagnostic needs assessments and utilization studies.

Where comprehensive, international initiatives do exist to document health and laboratory services, they are focused on the public sector.[12] This selective focus is not ideal as a significant portion of health care in resource-limited settings is delivered outside of the public sector. The World Bank estimates that 50.5% of medical expenditures in sub-Saharan Africa are delivered through the private sector.[13] Similarly, Elbireer *et al*. showed that 96% of laboratories in Kampala were private and these private laboratories produced 65% of the aggregate testing volume.[14]

This deficit of knowledge of the full landscape of laboratory testing outside of the public sector deprives policy makers, funders, and test developers of the ‘on the ground’ information they require to understand diagnostic needs. In an effort to address this knowledge deficit, we report on test identity, availability, and prices in Kampala, Uganda. The data derive from a comprehensive survey of clinical laboratories in Kampala in late 2011, including the public and non-public sectors.[15]

## Methods

A cross-sectional in-person survey of all clinical laboratories in Kampala, Uganda was performed during the last quarter of 2011. Details of the methods have been described previously.[15] Briefly, a 13-member survey team was divided into 5 groups of 2–3 individuals each. Each group was responsible for assessing each of the five geographic and administrative sub-divisions of Kampala city. This was achieved by visiting laboratories registered with the government as well as walking from street to street looking for laboratory locations. Survey questions were administered in person to the “in-charge” or designee at each laboratory facility. Clinical laboratories were defined as all establishments where laboratory tests are performed on human specimens for the purpose of health care. They included standalone laboratories (i.e., those not associated with a health care establishment), as well as those embedded within health care establishments. The Ministry of Health in Uganda supported and collaborated in the survey.

Laboratories were also classified according to affiliation as public, private, academic, or non-governmental organization (NGO)/religious laboratories. Academic laboratories were only those associated with Ugandan academia and not foreign academia. For example, a laboratory primarily doing research testing sponsored by western research universities would be classified as ‘private’, not academic. Surveyors also classified each laboratory as either a point-of-care (POC) laboratory or a moderate/high complexity laboratory (hereafter referred to simply as high complexity). Laboratories performing only single-step kit tests, single test instruments, or simple light microscope examinations of patient samples were classified as POC laboratories. Finally, laboratories were graded based on the Stepwise Laboratory Quality Improvement Process Toward Accreditation (SLIPTA) checklist, and graded on an ordinal system (0 to 5 stars).[16] Further details of the survey have been described in an earlier publication.[15]

### Test type and Test Prices

Test menus as well as laboratory-wide daily test volumes were documented in-person by the surveyors. For the purpose of this report, hard copies of test menus were requested from the 31 laboratories with greater than 20 tests performed daily. This specific request was made to minimize the impact of potential surveyor error. These additional data were successfully collected from 14 of the 31 larger laboratories in March, 2014. In addition, in April, 2015, prices for the most commonly offered tests were obtained from a sample of 20 private laboratories (8 POC and 12 high complexity, roughly mirroring the contribution to total testing volumes in Kampala by POC and high complexity laboratories, respectively).[17] Prices were adjusted to 2011 according to the World Bank estimates of Ugandan consumer price index.[14] Private laboratories were chosen as they are cash-based and thus represent the price of testing in the marketplace, with minimal price adjustments due to government programs and foreign aid. In our analysis we treated reported laboratory-wide test volumes as those of individual tests and not test panels.

Test menus were not uniform in terms of what individual tests were called. Some test names were based on the analyte, others on the related disease. Thus, in order to combine the data from different laboratories individual tests were combined into diagnostic disease *test types*, hereafter referred to simply as *tests*. For example, rapid diagnostic tests for malaria and blood smear microscopy for malaria were combined under the heading *Malaria*. On the other hand, some tests used for the same disease were kept separate, e.g., HIV serology, CD4 cell count, and HIV PCR. Furthermore, multiple formats of a test were combined into the same heading. For example, HIV serology tests could have been performed as a rapid lateral flow diagnostic or on an automated instrument, and there was no discrimination of whether tests labeled HIV PCR measured RNA or DNA. Also, when a laboratory did offer a panel we assumed it offered all of the individual components as well. For example, a laboratory offering a basic metabolic panel was treated as though it also offered electrolytes, renal function tests, and glucose. Ambiguous test names, e.g., “micro other”, were included in the analyses concerning testing volumes of each laboratory, but excluded from other analyses.

### Test Availability

One estimate for availability of a test is simply the number (or percent) of laboratories offering the test. But it is reasonable to assume that not all laboratories contribute equally to availability. For instance, a larger laboratory will be more likely to have reagents in stock and more likely to receive samples in blood collection tubes from clinics in a larger geographic area, increasing the population of patients they serve. To account for this, in most analyses instead of using the percent of laboratories that offered a test, we used the Availability Index, which weights the percent of laboratories that offered a test by the laboratory-wide test volumes of those laboratories. This index was calculated as the aggregate laboratory-wide daily testing volume of all laboratories offering a given test, divided by the aggregate laboratory-wide daily testing volume of all laboratories in Kampala, expressed as a percentage. The total daily testing volume for individual laboratories was for all tests offered in that laboratory and not just the daily test volume of the test of interest. For example, urinalysis was offered in 496 laboratories that together performed 10,720 tests daily (including all types of tests, not only urinalysis), and the total testing volume daily in Kampala (including all types of tests) was 13,189, therefore the urinalysis Availability Index is equal to 100*(10,720/13,189), or roughly 81%. The weighted percent of laboratories can range between 0 and 100% for each test.

Cluster analysis is a means of determining subgroups within data (in this case, test availability). *K*-means clustering was implemented in Stata 12. One-dimensional cluster analysis was performed on the Availability Index. To choose the number of clusters used in the *K*-means algorithm, we used the elbow method. Briefly, we plotted the sum of the within-group sum of squares as a function of the number of clusters used in the *K*-means algorithm (S1 Fig). The final number of clusters used in downstream analyses was chosen such that adding an additional cluster did not significantly explain more variance in the data.

In order to provide context to the price of tests in Kampala, we queried the Center of Medicare and Medicaid Service’s schedule of laboratory fees to compare the U.S. price of similar tests.[18] Test prices in the U.S. were calculated based on the mean prices of all test codes that corresponded to the test found in Kampala laboratories, weighted by the relative annual volumes of these different CMS test codes and adjusted to 2011 dollars by the consumer price index (S1 Table).

## Results

**Fig 1:**
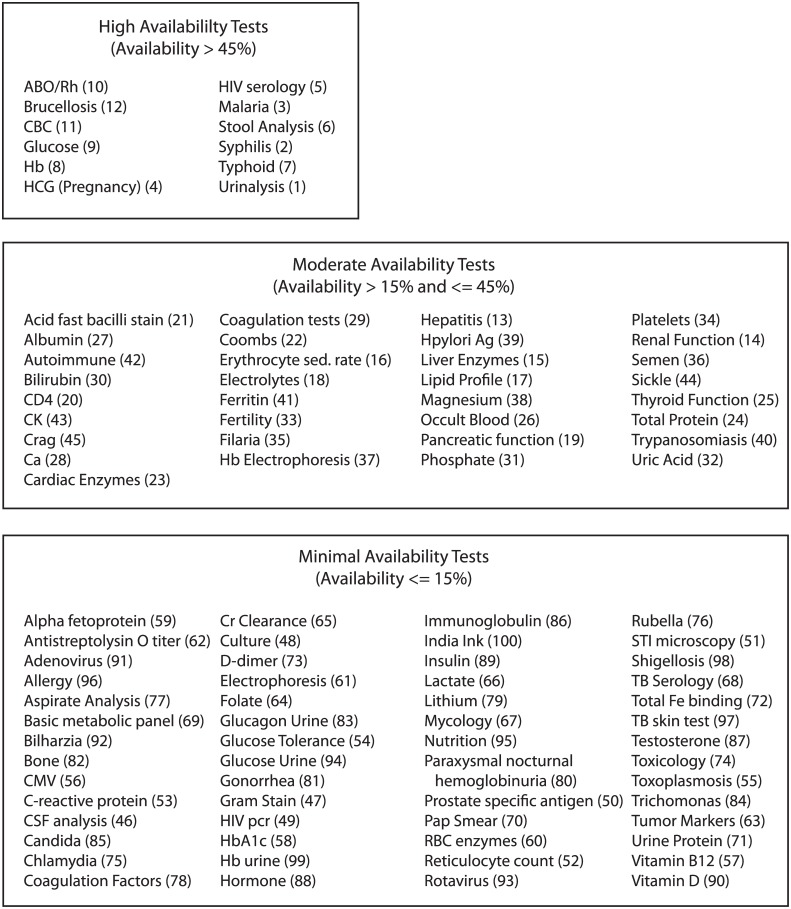
Full list of tests offered in Kampala, grouped by Availability Index. The numbers in parentheses refer to position of the test in Figs 2 and 3. The Availability Index takes into account the size of the laboratories offering the test. See methods for the calculation.

Test menus and daily laboratory-wide test volumes were obtained from 95% (907/954) of the laboratories identified in Kampala city. Based on these 907 laboratories, 100 different types of tests were offered in the city (Fig 1).

### Common Test Types and their Prices

Table 1 shows the 10 most commonly offered tests in Kampala. It also shows the average price of these tests in Kampala in Ugandan shillings and U.S. dollars, and the price of similar tests in the United States as determined by Center for Medicare and Medicaid Services clinical laboratory fee schedule.[18] In decreasing order, the most commonly offered test types in Kampala were Malaria, Human Chorionic Gonadotropin (HCG), HIV serology, Syphilis, Typhoid, Urinalysis, Brucellosis, Stool Analysis, Glucose, and ABO/Rh typing. The average price of these tests in Kampala, Uganda was $2.62, ranging from $1.83 to $3.46. In the U.S., the average price of similar tests was $10.21, ranging from $3.62 to $17.97.

**Table 1:** Ten most commonly offered tests in Kampala: Names, Number of laboratories, and Prices.

Test	Number of Labs	Percent of Labs	Price per test in Kampala (Uganda Shilling)*	Price per test in Kampala (US$)**	Price per test in US (US$)***
**Malaria** ****	822	91%	5,321	1.83	7.95
**HCG**	743	82%	6,375	2.20	11.24
**HIV**	736	81%	8,887	3.06	17.97
**Syphilis**	619	68%	7,940	2.74	7.00
**Typhoid**	560	62%	9,838	3.39	17.27
**Urinalysis**	496	55%	7,561	2.61	3.62
**Brucellosis**	353	39%	10,041	3.46	11.70
**StoolAnalysis**	350	39%	6,965	2.40	17.22
**Glucose**	293	32%	5,937	2.05	4.19
**ABORh**	228	25%	7,298	2.52	3.90

* Prices adjusted by the Ugandan consumer price index to 2011 prices

** Prices converted at an exchange rate of 2,900 Ugandan Shillings to 1 US dollar

***Prices derived from CMS fee schedule (see methods) and converted to 2011 prices

****Price is for malaria smear microscopy.

### Test Availability

**Fig 2:**
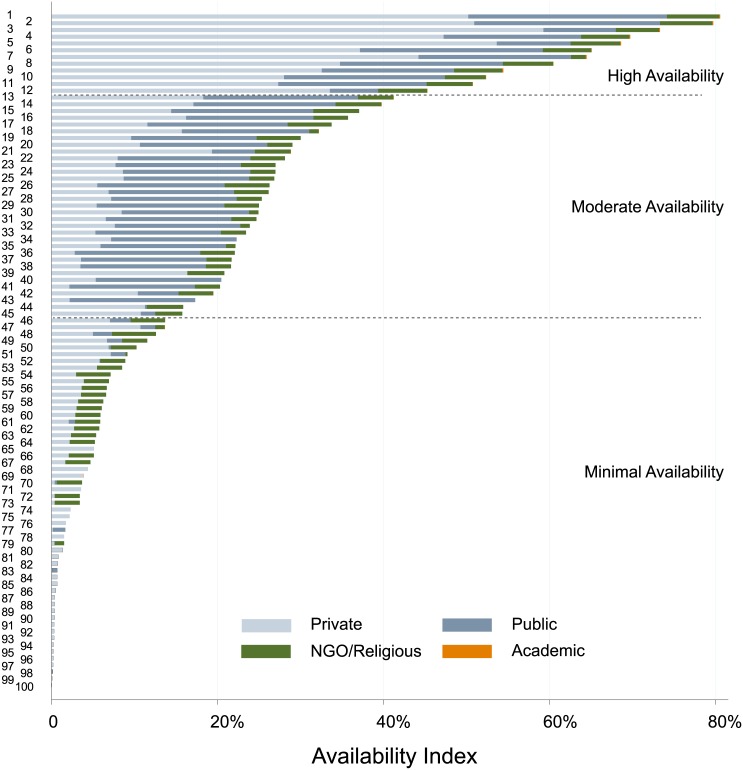
Bar graph of tests offered in Kampala, ranked by Availability Index* and sub-categorized by laboratory affiliation. Bar graph depicting the contribution of different sectors in the laboratory market to availability of tests in Kampala, Uganda. Each number on the Y axis corresponds to a different test (see Fig 1 or S2 Table for identification of tests).

**Fig 3:**
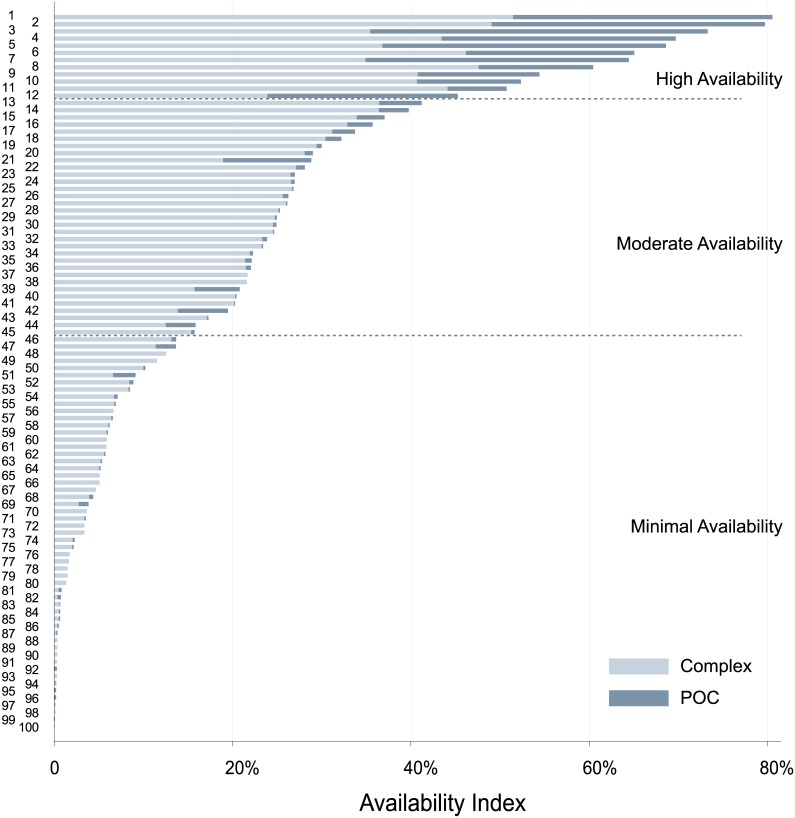
Bar graph of tests offered in Kampala, ranked by Availability Index* and sub-categorized by laboratory complexity. Bar graph depicting the contribution of complex versus POC laboratories to availability of tests in Kampala, Uganda. Each number on the Y axis corresponds to a different test (see Fig 1 or S2 Table for identification of tests).

Tests varied widely in their availability. The 10 most commonly offered tests were offered in a median of 528 laboratories each, while the bottom 80 tests were offered in a median of 6 laboratories each. The 100 tests offered in Kampala clustered into 3 groups based on their Availability Indices (S1 Fig). The full bars in Figs 2 and 3 represent the raw data points used in clustering. The boundaries (dashed lines) describing high, moderate, and minimal availability represent results of clustering. The three test clusters had average Availability Indices of 64%, 26%, and 4%, which we describe as high, moderate, and minimal availability (Fig 1). The high availability group consisted of 12 types of tests, the moderate availability group consisted of 33 types of tests, and the minimally available group consisted of 55 types of tests.

### Laboratory Affiliation

Private laboratories offered 99 different types of tests, public laboratories offered 56 types of tests, religious/NGO laboratories offered 66 types of tests, and academic laboratories offered 19 types of tests.

As the vast majority of laboratories in Kampala are private laboratories,[15] the availability of a given test as measured solely by the number of laboratories offering the test, is dominated by the private sector (Table 1). However, when using the Availability Index, which adjusts for the size of laboratories offering the test, both private and public laboratories contributed substantially (Fig 2). Private laboratories contributed 65% and public laboratories contributed 26% to the availability of highly available tests. Public laboratories contributed 52% and private laboratories contributed 35% to the availability of moderately available tests. Private laboratories contributed 59% and NGO/religious laboratories contributed 34% to the availability of minimally available tests. Overall, test availability was provided primarily through private laboratories (50%) and public laboratories (36%). Tests in which availability was provided largely by private laboratories included tests that are available in POC formats (e.g. HIV serology) as well as those not available in POC formats (e.g. HIV PCR). Conversely, tests with availability largely deriving from public laboratories tended to be tests that are not typically available in POC formats (e.g. thyroid function, bilirubin). Finally, private laboratories offered 18 tests in excess of 15% Availability Index, while public laboratories offered 37 such tests.

### Laboratory Complexity

Laboratories in the survey were also categorized based on whether they performed only simple POC tests, or also high complexity tests (Fig 3). POC laboratories contributed significantly only to a handful of tests, but these ranked among the most available in Kampala. The high availability group of tests had significant contribution from both POC and high complexity laboratories. The moderate and minimal availability groups of tests had significant contribution only from high complexity laboratories.

### Spending on Laboratory Testing

The average price of a test in Kampala was $2.62 (Table 2) and the aggregate daily testing volume of all laboratories in Kampala, Uganda was 13,189. Assuming 250 active days per year for laboratories, this equates to 3.3 million tests annually. The population of Kampala is estimated at 1.72 million,[19] and thus 1.9 tests per person per year were performed on average, and at $2.62 per test, $5.02 was spent on testing per person per year. The World Bank estimates the amount spent on health expenditures per person per year in Uganda was $54 in 2011.[17] Because Kampalans likely spend more on healthcare per person than Ugandans, this $54 was multiplied by an estimate of the ratio of consumption expenditure in Kampala vs Uganda. According to the Ugandan Board Of Statistics in 2012, this ratio was 2.0 when considering the monthly household consumption expenditure per month and was 2.8 when considering the average per capita consumption expenditure.[19] Depending on which estimate is used, the annual spent on health per person in Kampala ranges from $110 to $152. If $5.02 is spent on testing per person per year in Kampala, this estimate of medical laboratory expenditures represents 3.3% to 4.6% of total health spending.

**Table 2:** Per capita spending on Laboratory Testing in Kampala, Uganda.

Health Spending on Laboratory Testing in Kampala, Uganda				
Average price of a test	Annual tests per person*	Annual $ spent on tests per person	Annual $ spent on health per person**	Health spending on laboratory
$2.6	1.9	$5.0	$110–$152	3.3%–4.6%

* Assumed population of Kampala of 1.72 million[19]

**Annual total health spending per person for Uganda taken from The World Bank, adjusted by the ratio of consumption expenditures in Kampala vs Uganda (see methods)[19].

## Discussion

This report, based on a comprehensive survey of laboratories in Kampala, Uganda (population ~ 1.72 million), presents data on test availability and price in this sub-Saharan African city. It also describes the relationship of test availability to laboratory affiliation, such as private, public, NGO/religious, or academic, as well as laboratory complexity. There were 100 distinct test types offered in Kampala. These tests fell into three groups in terms of their relative Availability Index (high, moderate, minimal) and the average prices of the 10 most commonly offered tests ranged from $1.83-$3.46.

### Number of Test Types in Kampala

This survey included all tests from both public *and* non-public laboratories. Compared to prior studies, this more comprehensive approach helped unearth unique information. For example we found 100 different types of tests in use in Kampala city, where the DHS Program Service Provision Assessment (SPA) typically surveys for roughly 25 tests.[12] Clearly, 100 is a larger number than 25, but it begs a deeper question: is this variety broad enough to address the basic clinical queries of Kampala’s citizens’? This question cannot be answered directly because local disease burdens in Kampala are not well-described, and furthermore, refined test utilization algorithms do not exist for most tests. However an indirect comparison, based on patterns of laboratory testing use in the USA, suggests that 100 tests are sufficiently varied to meet the basic clinical needs of a community. To illustrate, there are 1250 Healthcare Common Procedure Coding System (HCPCS) codes listed in the CMS clinical laboratory fee schedule.[18] However, the CMS Physician Supplier Procedure Summary 2012 data base, which lists tests and volumes reimbursed by CMS programs, shows that 100 HCPCS codes account for 86% of test volume, and 200 codes account for 95% of test volume. Furthermore, a particular type of test may be represented in more than one code. For example, there is an HCPCS code for manual urinalysis, and another for automated urinalysis. Seen in this context, the 100 types of tests available in laboratories in Kampala appear to provide a level of test variety that is on par with the basic level of test variety in Western countries.

However, this conclusion assumes that all 100 tests are available at a level needed for clinical care. Although disease burdens do vary between Kampalan and U.S. populations, overall testing per person in Kampala is 2 tests per person per year, contrasted with 20 to 30 tests per person per year in the United States.[20,21] Furthermore, the availability of roughly half of the 100 tests found in Kampala—those in the minimal availability cluster—was very restricted. To illustrate, at the time of the survey we found only 2 laboratories in Kampala providing D-dimer testing. Further studies investigating disease burdens and optimal test utilization could help address if test availability is adequate.

### Test Availability versus Disease Burden

Test availability appears to follow burden of disease. For example, HIV and malaria rapid testing are highly available, while testing for prostate-specific antigen and testosterone are minimally available. Still, there are some peculiarities that deserve discussion.

First, although tuberculosis represents a major burden of disease, tuberculosis testing does not appear in the high availability group. There are likely explanations for this. For example tuberculin skin testing (TST), an inexpensive screen for TB, is minimally available. While this may not seem rational at first, Uganda recommends use of BCG vaccination; thus reducing the utility of TST. Likewise, tuberculosis serological testing is in the minimal availability group, but this test has not proven to be useful for tuberculosis testing and as discussed below, the World Health Organization has recommended against its use. Acid fast bacilli staining and microscopy is an appropriate testing strategy for Kampala, but is only found in the moderate availability group. This is likely because it is a complex test while the high availability testing group consists nearly exclusively of tests that are available in easy to use, point of care formats (see Fig 3). This gap supports the global effort that has been underway to develop and employ inexpensive point of care devices for tuberculosis testing.[22]

Second, tests for non-communicable diseases, with the exception of glucose, were found in the moderately available group. This is likely because all these tests (except glucose) are offered nearly exclusively in complex laboratories while glucose testing is offered in POC labs as well as complex labs (Fig 3).

Finally, some tests addressing relatively low burdens of disease are much more available than many tests addressing high burdens of disease. This can be understood in part by realizing that the ideal availability of a test depends on many factors beyond the burden of disease it addresses: cost of the test, accuracy of the test, clinical impact of the test (e.g., does it change management and outcome), and use of the test in diseases that mimic more common diseases with higher burdens. For example, several accurate, inexpensive, and easy to use tests are available for syphilis testing (Treponema pallidum particle agglutination assay, rapid plasma reagin, and multiple lateral flow diagnostic immunoassays), which may explain why syphilis testing is the 2nd most available test although it addresses a relatively low burden of disease. Likewise, brucellosis testing is relatively highly available while addressing a relatively low burden of disease. This could be due to availability of inexpensive lateral flow testing formats, high prevalence of brucellosis in Uganda with occasional outbreaks, and the fact that brucellosis is on the differential with more common non-specific febrile disease like malaria.[23]

### Test Availability versus Laboratory Affiliation

Only public and private laboratories contributed significantly to the availability of high and moderate availability tests. Private and NGO/religious sectors were the primary contributors of the minimal availability tests, thus providing low volume, niche tests like Hb A1c, C-reactive protein, and HIV PCR. For example, contributions to the HIV PCR Availability Index were 58% from private laboratories, 26% from NGO/religious laboratories, and 16% from public laboratories. This pattern is consistent with recent literature demonstrating the significant impact the private sector has on health care in resource limited settings. For instance, The World Bank estimates that 74% of health care dollars spent in Uganda are spent in the private sector, and 50.5% of health care dollars spent in sub-Saharan Africa are spent in the private sector.[13]

However, the impact of private sector health care delivery is not always positive. The World Bank reported that the private health sector was regulated as intended in only 6 of 45 countries.[13] To illustrate the impact that an unregulated laboratory testing market can have, we present the example of tuberculosis serology testing in India.[24] TB serology testing has never enjoyed a robust evidence base for diagnosis of TB. For this reason the WHO had never included these tests in guidelines. However, the private sector in India primarily offered serological TB tests because they were very popular among patients and physicians.[25] Subsequent studies showing lack of diagnostic utility were performed[26,27] and led the WHO to issue their first ever negative endorsement.[28] Today, TB serology testing is no longer legal in India. This example shows the negative impact the private sector can have, operating largely out of view of policy makers. Because of the significant impact the private sector makes to many health systems, it should not be ignored.

### Test Availability versus Test Complexity

Regarding POC vs high complexity laboratory testing, our data are consistent with the prevailing idea that implementation of POC diagnostics can increase test access in resource-limited settings.[2,29] In our survey, tests that had the greatest Availability Indices were also those that were being offered to a significant extent in POC laboratories, and were thus available in a POC format. Roughly one-third of the availability of the high availability tests were contributed by POC laboratories. Of the moderate and minimal availability tests, only 6% was contributed by POC laboratories.

But a key concern of promoting additional POC testing is quality. Although there are very few laboratories of any type in sub-Saharan Africa that are accredited to international standards,[30] smaller laboratories in both high- and low-resourced environments, tend to be more POC-reliant and more quality-challenged than larger laboratories.[31] The smaller laboratories in Kampala, which were largely POC laboratories, tended to be the laboratories with the lowest quality scores. Elbireer *et al*. survey found that 704 out of 718 POC laboratories did not meet the lowest quality standards defined by the WHO/AFRO-derived laboratory strengthening tool (1–5 stars).[15] Thus the benefits of the POC format must be balanced against the challenge of assuring quality in the laboratories that will likely be using the tests.

### Test Prices

Finally, our data show that the most commonly offered tests are priced between $1.83 and $3.46. We estimate that a similar fraction of the household healthcare budgets in Kampala is spent on laboratory testing compared to levels in the US market. At a population level of 1.72 million and a daily testing volume of 13,189, citizens of Kampala, Uganda consume an average of 1.9 tests per year. In the United States this number is estimated at 20 to 30 tests per person per year,[20,21] which is clearly much higher. However, when considering the health dollars spent in Kampala and the price of testing, total investment in laboratory medicine is substantial. As detailed in Table 2, our estimates of the percentage of health spending in Kampala that is devoted to laboratory testing ranges from 3.3% to 4.6%. This rough estimate is similar to that for the United States where 3% of Medicare part B payments in 2010 were laboratory expenditures.[32] Furthermore, estimates of U.S. laboratory testing expenditures as a percent of total health expenditures have been very stable, varying between 2–3% from 1998 to 2007.[20]

This analysis had several limitations. First, the survey was conducted in 2011 and there may have been significant changes in the laboratory landscape since that time. For example, if more automated analyzers are being employed in Kampala, it would be likely that testing for non-communicable diseases—which are more commonly performed on automated analyzers—has increased. Second, the original survey only collected up to 18 tests per laboratory. Although this was sufficient for the majority of laboratories[14] some did offer more than 18 tests and in the attempt to gather these additional data we were successful for only a subset of laboratories. Third, price data were collected in 2015, and although adjusted by the consumer price index, may have introduced a bias when comparing to the original 2011 test volume data. Furthermore, price data was collected from a small subset of the laboratory landscape (20 laboratories). Although they were chosen to represent the complexity distribution of the overall laboratories, they may not represent the full variation of prices in Kampala. Additionally, test menus and volumes were self-reported as opposed to employing more active surveillance methods such as review of monthly testing workloads. Still, this study is important because it represents the first comprehensive description of the utilization of laboratory testing in a large sub-Saharan Africa city. It provides unique actionable data to guide interventions that are locally relevant.

## Supporting Information

S1 Data Availability (ZIP)

S1 Fig Elbow curve of variance explained by number of clusters. (DOCX)

S1 Table Data used to calculate test type prices from the CMS laboratory fee schedule. (DOCX)

S2 Table Identification of tests in Figs 2 and 3. (DOCX)
