# Supplementary material for: Laboratory Diagnostics Market in East Africa: A Survey of Test Types, Test Availability, and Test Prices in Kampala, Uganda
Source: PLoS One. 2015 Jul 30;10(7):e0134578. doi: 10.1371/journal.pone.0134578 (PMC4520457; doi:10.1371/journal.pone.0134578)
Supplement: S1 Data Availability — (ZIP) [file pone.0134578.s001.zip › DataAvailabilityCaptions.docx]

**TestTypes_data_availability.csv**

This file includes primary data regarding the types of tests offered in the 907 laboratories in Kampala, Uganda from which we were able to collect test type data. The first row is a header with descriptions of each column.

LabIdentifier: an arbitrary laboratory code

DailyTestingVolume: daily testing volume for all tests in a laboratory

Affiliation: the affiliation of the laboratory (Public, Private, NGO/Religious, Academic)

Complexity: whether a lab is a laboratory offering only simple, point of care tests or also moderate/high complexity tests

Test1 – Test73: the menu of tests offered at a given laboratory

**TestPrices_data_availability.csv**

This file includes primary data regarding the prices of tests offered in 20 laboratories in Kampala, Uganda from which we were able to collect data. The first row is a header with descriptions of each column.

LabID: an arbitrary laboratory code

Columns 2-11: price in U.S. dollars for the test (see column name for type of test)

Complexity: whether a lab is a laboratory offering only simple, point of care tests or also moderate/high complexity tests
