# Supplementary material for: Laboratory Diagnostics Market in East Africa: A Survey of Test Types, Test Availability, and Test Prices in Kampala, Uganda
Source: PLoS One. 2015 Jul 30;10(7):e0134578. doi: 10.1371/journal.pone.0134578 (PMC4520457; doi:10.1371/journal.pone.0134578)
Supplement: S1 Fig — (DOCX) [file pone.0134578.s002.docx]

**S1 Fig. Elbow curve of variance explained by number of clusters.**


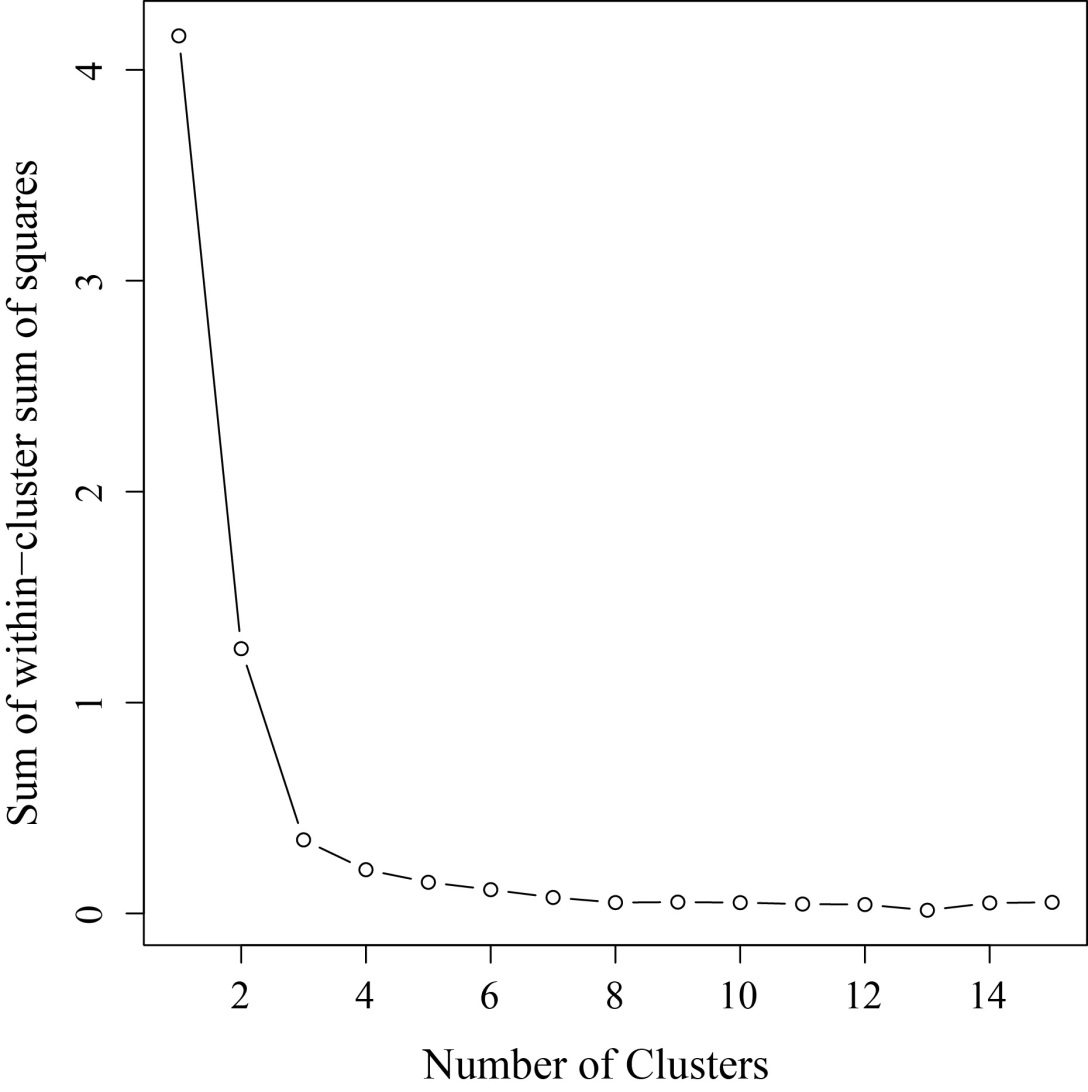


Supplementary Figure 1: *K*-means clustering analysis was used to categorize tests in terms of availability as defined by the Availability Index (see methods and Figure 1). The number of clusters to use in *K*-means analysis was determined by plotting the sum of the within-group (within-cluster) sum of squares as a function of increasing numbers of clusters (1-15 clusters; 3 clusters was sufficient to explain most of the variation).
