# Supplementary material for: Laboratory Diagnostics Market in East Africa: A Survey of Test Types, Test Availability, and Test Prices in Kampala, Uganda
Source: PLoS One. 2015 Jul 30;10(7):e0134578. doi: 10.1371/journal.pone.0134578 (PMC4520457; doi:10.1371/journal.pone.0134578)
Supplement: S1 Table — (DOCX) [file pone.0134578.s003.docx]

**S1 Table. Data used to calculate test type prices from the CMS laboratory fee schedule.**

| **HCPCS** | **Test Type (Kampala)** | **HCPCS description** | **CMS National Limit ($)*** | **# tests through CMS (2012)** |
| --- | --- | --- | --- | --- |
| 86900 | ABORh | Blood typing abo | $3.90 | 56,446 |
| 86622 | Brucellosis | Brucella antibody | $11.70 | 1,998 |
| 82947 | Glucose | Assay glucose blood quant | $5.13 | 3,204,448 |
| 82950 | Glucose | Glucose test | $6.22 | 46,772 |
| 82962 | Glucose | Glucose blood test | $3.07 | 2,765,709 |
| 81025 | hCG | Urine pregnancy test | $8.29 | 95,609 |
| 84702 | hCG | Chorionic gonadotropin test | $19.71 | 37,026 |
| 84703 | hCG | Chorionic gonadotropin assay | $9.84 | 23,051 |
| 86701 | HIV | Hiv-1antibody | $11.63 | 8,704 |
| 86702 | HIV | Hiv-2 antibody | $17.70 | 2,307 |
| 86703 | HIV | Hiv-1/hiv-2 1 result antbdy | $17.95 | 183,778 |
| 87389 | HIV | Hiv-1 ag w/hiv-1 & hiv-2 ab | $31.53 | 4,150 |
| 87390 | HIV | Hiv-1 ag eia | $23.10 | 553 |
| 87391 | HIV | Hiv-2 ag eia | $23.10 | 121 |
| 86750 | Malaria | Malaria antibody | $17.27 | 95 |
| 87207 | Malaria | Smear special stain | $7.85 | 8,387 |
| 87177 | Stool Analysis | Ova and parasites smears | $11.65 | 177,661 |
| 87209 | Stool Analysis | Smear complex stain | $23.53 | 156,536 |
| 86592 | Syphilis | Syphilis test non-trep qual | $5.59 | 358,943 |
| 86593 | Syphilis | Syphilis test non-trep quant | $5.76 | 15,190 |
| 86780 | Syphilis | Treponema pallidum | $17.33 | 50,809 |
| 87285 | Syphilis | Treponema pallidum ag if | $15.70 | 16 |
| 86768 | Typhoid | Salmonella antibody | $17.27 | 1,790 |
| 81000 | Urinalysis | Urinalysis nonauto w/scope | $4.14 | 3,496,795 |
| 81001 | Urinalysis | Urinalysis auto w/scope | $4.14 | 7,641,279 |
| 81002 | Urinalysis | Urinalysis nonauto w/o scope | $3.35 | 4,871,013 |
| 81003 | Urinalysis | Urinalysis auto w/o scope | $2.94 | 6,348,688 |
| 81005 | Urinalysis | Urinalysis | $2.84 | 227,699 |
| 81015 | Urinalysis | Microscopic exam of urine | $3.98 | 199,554 |
| 81020 | Urinalysis | Urinalysis glass test | $4.83 | 3,210 |

* CMS prices are adjusted by the consumer price index from 2013 to 2011.
