# Supplementary material for: Laboratory Diagnostics Market in East Africa: A Survey of Test Types, Test Availability, and Test Prices in Kampala, Uganda
Source: PLoS One. 2015 Jul 30;10(7):e0134578. doi: 10.1371/journal.pone.0134578 (PMC4520457; doi:10.1371/journal.pone.0134578)
Supplement: S2 Table — (DOCX) [file pone.0134578.s004.docx]

**S2 Table. Identification of tests in Figs. 2 and 3.**

| **Test Types** | | | | | | | | | | | | |
| --- | --- | --- | --- | --- | --- | --- | --- | --- | --- | --- | --- | --- |
| 1 | Urinalysis | | 26 | | | Occult Blood | | 51 | STI microscopy | | 76 | Rubella |
| 2 | Syphilis |  | | 27 | | Albumin |  | 52 | Reticulocyte count |  | 77 | Aspirate Analysis |
| 3 | Malaria |  | | 28 | | Calcium |  | 53 | C-reactive protein |  | 78 | Coagulation Factors |
| 4 | HCG |  | | 29 | | Coagulation tests |  | 54 | Glucose Tolerance | | 79 | Lithium |
| 5 | HIV serology |  | | 30 | | Bilirubin |  | 55 | Toxoplasmosis | | 80 | PNH* |
| 6 | Stool Analysis | | | | 31 | Phosphate | | 56 | CMV |  | 81 | Gonorrhea |
| 7 | Typhoid |  | | 32 | | Uric Acid |  | 57 | Vitamin B12 |  | 82 | Bone |
| 8 | Hb |  | | 33 | | Fertility |  | 58 | HbA1c |  | 83 | Glucagon Urine |
| 9 | Glucose |  | | 34 | | Platelet |  | 59 | Alpha Fetoprotein |  | 84 | Trichomonas |
| 10 | ABO/Rh |  | | 35 | | Filaria |  | 60 | RBC enzyme | | 85 | Candida |
| 11 | CBC |  | | 36 | | Semen |  | 61 | Electrophoresis | | 86 | Immunoglobulin |
| 12 | Brucellosis | | 37 | | | Hb Electrophoresis | | 62 | Antistreptolysin O titer |  | 87 | Testosterone |
| 13 | Hepatitis |  | | 38 | | Magnesium |  | 63 | Tumor Markers | | 88 | Hormone |
| 14 | Renal Function | | 39 | | | H pylori Ag | | 64 | Folate |  | 89 | Vitamin D |
| 15 | Liver Enzymes |  | | 40 | | Trypanosomiasis | | 65 | Creatinine Clearance | | 90 | Insulin |
| 16 | Erythrocyte sed. rate |  | | 41 | | Ferritin |  | 66 | Lactate |  | 91 | Bilharzia |
| 17 | Lipid Profile | | 42 | | | Autoimmune | | 67 | Mycology | | 92 | Rotavirus |
| 18 | Electrolytes | | 43 | | | Creatinine Kinase |  | 68 | TB Serology | | 93 | Adenovirus |
| 19 | Pancreatic FCN | | 44 | | | Sickle cell test |  | 69 | Basic metabolic panel |  | 94 | Glucose Urine |
| 20 | CD4 |  | | 45 | | Cryptococcal antigen |  | 70 | Pap Smear | | 95 | Nutrition |
| 21 | Acid fast bacilli stain |  | | 46 | | CSF analysis |  | 71 | Urine Protein | | 96 | Allergy |
| 22 | Coombs |  | | 47 | | Gram Stain | | 72 | Total Fe binding capacity |  | 97 | Tuberculin skin test |
| 23 | Cardiac Enzymes | | 48 | | | Culture |  | 73 | D-dimer |  | 98 | Shigellosis |
| 24 | Total Protein | | 49 | | | HIV PCR |  | 74 | Toxicology | | 99 | Hb Urine |
| 25 | Thyroid Function | | 50 | | | Prostate Specific Ag |  | 75 | Chlamydia | | 100 | India Ink stain |

*Paroxysmal nocturnal hemoglobinuria
